# Balancing pH and Pressure Allows Boosting Voltage and Power Density for a H_2_–I_2_ Redox Flow Battery

**DOI:** 10.1021/acsaem.4c03032

**Published:** 2024-12-24

**Authors:** Kaustub Singh, Ameya Bondre, Kostadin V. Petrov, David A. Vermaas

**Affiliations:** Department of Chemical Engineering, Delft University of Technology, Van der Maasweg 9, 2629HZ Delft, The Netherlands

**Keywords:** redox flow battery, hydrogen, iodine, gas diffusion electrode, pH, crossover

## Abstract

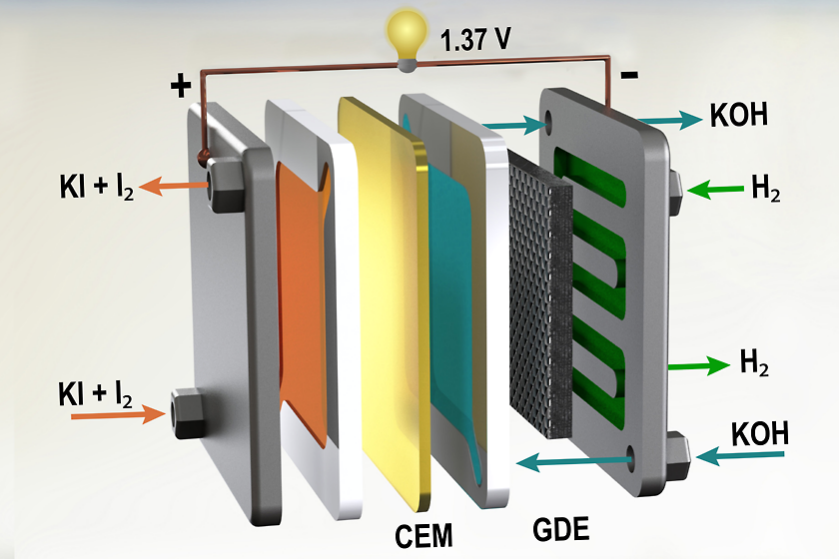

The decoupled power and energy output of a redox flow battery (RFB) offers a key advantage in long-duration energy storage, crucial for a successful energy transition. Iodide/iodine and hydrogen/water, owing to their fast reaction kinetics, benign nature, and high solubility, provide promising battery chemistry. However, H_2_–I_2_ RFBs suffer from low open circuit potentials, iodine crossover, and their multiphase nature. We demonstrate a H_2_–I_2_ operation with a combined neutral-pH catholyte (I_3_^–^/I^–^) and an alkaline anolyte (KOH), producing an open circuit cell voltage of 1.28 V. Additionally, we incorporate a pressure-balanced gas diffusion electrode (GDE) to mitigate mass transport limitations at the anode. These improvements result in a maximum power density of 230 W/m^2^ when allowing a mild breakthrough of H_2_ through the GDE. While minimal crossover occurs, side reactions of permeating active species were found reversible, enabling long-term operation. Future work should address the stability of the GDE and optimization of the electrolyte thickness and concentration to fully leverage the potential unlocked by balancing the pressure and pH in the H_2_–I_2_ RFB.

## Introduction

Redox flow batteries (RFBs) have attracted attention as a key contributor in the global effort to transition away from fossil fuels.^1−5^ RFBs have the ability to decouple energy and power output, implying that long-duration energy storage is possible without increasing the power capacity. This decoupling is possible by storing the electrochemically active redox electrolytes in reservoirs outside the power unit, unlike the commercial Li-ion batteries.^6,7^

Given the significance of the electrolytes to RFB performance, it is imperative to explore new redox chemistries and improve existing ones. Although over 50 redox couple combinations have been proposed in aqueous and organic media,^8,9^ very few combinations have been scaled to practical flow batteries as they struggle to fulfill the criteria of low cost, fast kinetics, high energy density, and safety.^10,11^ Iodine is one of such species, with applications in solar cells as well,^12^ due to its favorable characteristics including benign nature, fast reaction kinetics, and high solubility of the I^–^/I_3_^–^ redox couple.^13,14^ However, iodine-based flow batteries have not been developed to scale because (1) their standard redox potential is relatively close to standard hydrogen evolution potential (*E*^0^ = 0.54 V vs SHE), (2) iodine is poorly soluble in water, and (3) combining the gas phase of H_2_ with a liquid catholyte is challenging. In this work, we address this challenge by using H_2_ + KOH as an alkaline anolyte and I^–^/I_3_^–^ as a catholyte. By exploiting the pH dependency of the hydrogen reaction, an open circuit voltage of 1.37 V should be obtained at pH 14. Here, we demonstrate that we can run a system with stable pH when using an alkaline anolyte while keeping a pH-neutral catholyte.

The redox couples constituting the battery undergo the following reactions^15^
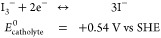
1at the cathode and
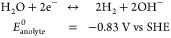
2at the anode. The pH of the anolyte is 14 to maximize the half-cell potential of H_2_/H_2_O. Such pH dependence does not exist for the catholyte.

Previous work on iodine-based flow batteries includes metal anodes such as Zn,^16^ Fe,^17^ Li,^18^ Al,^19^ and Mg,^20^ among others.^21,22^ These anodes have been proposed with I_2_ immobilized in microporous carbon to prevent I_3_^–^ from migrating to the anolyte. However, these combinations are compromised by either dendrite formation or surface passivation to a varying degree.^23^ Furthermore, these systems are limited by the amount of metal at the anode and thus do not have decoupled power and energy.

In a recent study, H_2_ was proposed as an anode candidate due to its high oxidation potential in alkaline media and fast kinetics.^23^ This H_2_–I_2_ chemistry has been explored in combination with bromide electrolytes^24^ and in a static H_2_–I_2_ cell.^23^ However, this static system has coupled power and energy due to the confinement of the I_2_ in microporous carbon. The other systems, with bromine-based electrolytes, can achieve high power density, but their corrosiveness and self-discharge present major concerns.^25^ While the kinetics and stability of the iodine chemistry have been shown to be promising, mass transfer and ion crossover in a flow battery concept remain unexplored.

To the best of our knowledge, we present for the first time a method to overcome the H_2_ mass transfer and crossover of iodine species for H_2_–I_2_ flow batteries under alkaline conditions. In this study, we present the electrochemical performance of hydrogen and iodine half-reactions representing a H_2_–I_2_ redox flow battery. We also validate our findings in a nonoptimized H_2_–I_2_ redox flow battery, demonstrating its potential for decoupled energy and power in long-duration energy storage.

## Materials and Methods

### Electrolyte Preparation

The catholyte solution was prepared by adding 1 M I_2_ (7553-56-2, Sigma-Aldrich, Germany) and 2 M KI (7681-11-0, Fischer Chemicals, The Netherlands) in a 9:1 (v/v) H_2_O/CH_3_CN (75-05-8, company) solution. The volume of catholyte used was 40 mL. The pH of the resulting solution was between 6.5 and 7.5. Although this concentration corresponds to just a modest 37 W h/L, the solubility of the polyiodide redox couples (>6 M) theoretically allows for energy densities exceeding 300 W h/L,^13,26^ which surpasses that of bromine-based flow batteries and is even competitive to alkali-ion batteries.^27−29^ To prevent the formation of insoluble I_2_ at high state-of-charge (SOC),^16^ we use a 90% water/10% acetonitrile (CH_3_CN) mixture. This mixture extends the solubility limits of I_2_ and KI to 5 and 10 M, respectively. The use of a mixed solvent finally makes the use of solid-based I_2_ species, as used in prior literature,^30^ obsolete and allows for utilizing the full potential of a H_2_–I_2_ flow battery with high energy density. Optimization of the water/acetonitrile ratio, or using other electrolytes or complexing agents,^31^ could be studied later to further enhance the energy density and stability.

A 1 M solution (40 mL) of KOH (1310-58-3, Emsure, Germany) was used as the anolyte. Hydrogen gas (Linde, Germany) was supplied in the gas compartment of the anode.

### Electrochemical Cell Assembly

**Figure 1:**
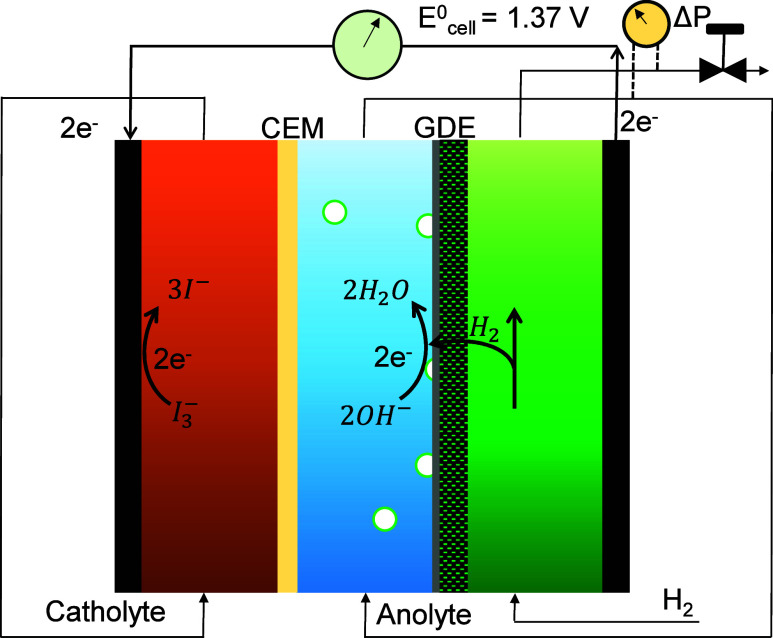
Schematic of a H_2_–I_2_ flow cell. The catholyte on the left, flowing against the Pt-coated titanium plate electrode, comprised a 2 M KI + 1 M I_2_ solution containing 10% CH_3_CN. A 1 M KOH anolyte, flowing against the Pt catalyst layer, was separated from the catholyte by a CEM. The H_2_ gas was fed to the cell along the carbon fiber substrate of the GDE. The H_2_ flow rate was controlled via a mass flow controller, and the pressure, relative to that of the anolyte, was modulated via a needle valve.

The H_2_–I_2_ RFB was conceptualized as a three-compartment cell, as shown in Figure 1. A cation-exchange membrane (CEM) separates the liquids and prohibits the crossover of the iodide and hydroxide ions. Ideally, the system could benefit from an anion-exchange membrane (AEM) that is highly selective for OH^–^. Such a configuration would allow the creation of a membrane electrode assembly at the anode, eliminating the use of the anolyte and the respective Ohmic losses. However, as such a membrane does not exist, we have equipped our system with a CEM. The CEM not only blocks the iodide and OH^–^ but also avoids crossover of iodine species, which are present as I_3_^–^ complexes.

A two-compartment microcell (ElectroCell, Denmark) module was used as the base for this cell. The catholyte was a single-phase liquid channel. The anolyte compartment was split into two—one compartment for liquid KOH and one for the H_2_ flow—by a gas diffusion electrode (GDE). A Freudenberg GDE (SKU—1590035, Fuel Cell Store, USA), sputtered with a Pt catalyst over the anolyte-facing side, was used to partition the anolyte and the H_2_ gas. Ag/AgCl microreference electrodes were inserted at the center of the electrolyte flow channels, adjacent to the membrane.

The anolyte and the catholyte were pumped through the 3 mm thick flow channels at 60 mL/min and were separated by a 75 μm thick CEM (Fumasep FKB-PK-75, Germany). This membrane allows both cations and protons but blocks the OH^–^ and iodide/tri-iodide species, with a high counter-/co-ion selectivity (96–99% according to the specs). Given the neutral or high pH in our system, we expect K^+^ to be the dominant charge carrier through the CEM. An estimate, based on the initial concentrations and membrane selectivities, shows that no significant H^+^ crossover is expected and slight OH^–^ crossover during the charging phase (see Supporting Information Table S2).

The H_2_ gas flow rate was fixed at 80 mL/min and controlled via a mass flow controller (Bronckhorst, The Netherlands). A needle valve (Swagelok SS-SS6MMVH, USA) was placed at the gas outlet to control the pressure of H_2_, and the gas back pressure, relative to the anolyte, was measured with a differential pressure transmitter (Endress + Hauser PMD75, Switzerland).

To highlight the overall gain of adding a GDE to the H_2_–I_2_ cell, a two-compartment control experiment was performed. The H_2_ in this experiment was sparged into the anolyte reservoir and carried into the cell in the dissolved state. A schematic illustrating this system is presented in Figure S1a.

To assess the performance of the H_2_–I_2_ cell for varying pressure balancing and pH conditions, we performed linear sweep voltammetry (LSV) and chronopotentiometry to assess the power density and cycling behavior. Experimental details are in Supporting Information.

## Results and Discussion

The anodic operation half-cell reaction requires reactants in both liquid (KOH) and gas phase (H_2_) to react on the solid catalyst (Pt) surface.^32^ The use of an alkaline anolyte in this configuration allows for the use of more earth-abundant catalysts. The necessity of a gas compartment is highlighted when running the battery in a two-compartment setup, with the catholyte and the H_2_-sparged anolyte flowing on either side of the CEM (Figure S1a). For this system, the OCV was measured at 1.28 V, close to the expected 1.37 V (eqs 1 and 2), but the cell voltage dropped to 50% of its OCV within 2 min when discharging at 5 A/m^2^ (Figure S1b). This indicated a mass transport limit within the cell. When the electrodes were assessed separately, the cathodic half-cell sustained current densities of up to 700 A/m^2^ until the half-cell voltage lost 50% of its initial OCV (Figure S2). Therefore, it was concluded that the H_2_ oxidation reaction (HOR) at the anode must suffer from a mass transport limitation when relying on dissolved H_2_. It has been previously shown that HOR becomes diffusion-controlled in strongly alkaline media, thus limiting the oxidation rate.^32^ The low solubility of H_2_ in 1 M KOH (0.08 mM) together with a small diffusion coefficient (5 × 10^–9^ m^2^/s)^33^ clearly limits this system.

As it became evident that increasing reactant supply to the catalyst surface is key in increasing HOR rate and, consequently, the overall power density of the cell, a Pt-sputtered Freudenberg H23C6 GDE was placed between the anolyte channel and the gas feed to enhance H_2_ transport to the catalyst surface without sparging. This specific GDE was used because of its exceptional resistance to flooding.^34^ In addition, a differential pressure meter was used between the H_2_ gas compartment and anolyte, and a needle valve was placed at the gas outlet to control the H_2_ breakthrough in the GDE.

**Figure 2:**
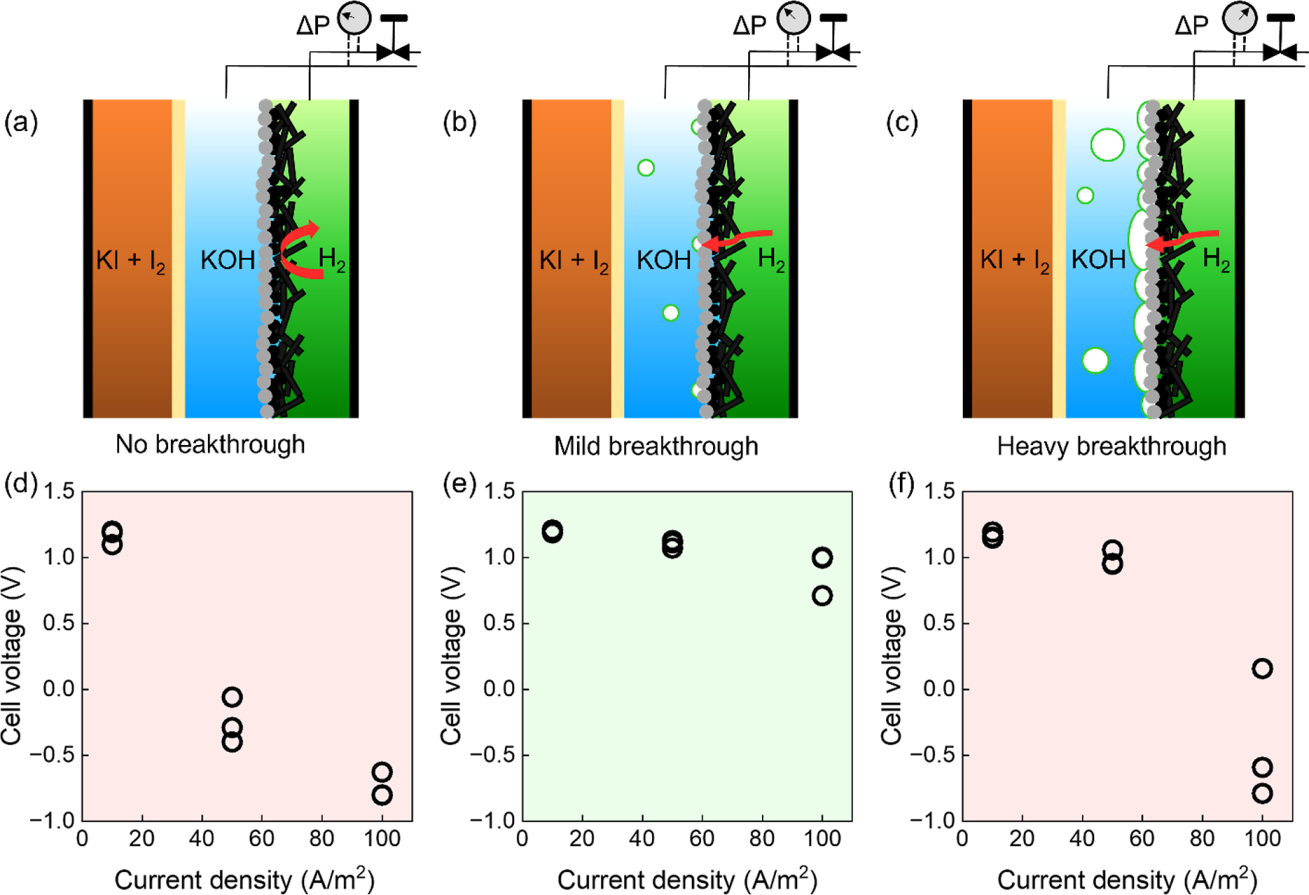
Flow regimes with (a) no, (b) mild, and (c) heavy breakthrough of H_2_ through the GDE into the anolyte (KOH). The penetration of H_2_ into the GDE is controlled by tuning the gas back-pressure via the needle valve placed at the gas outlet. Cell voltages at three different discharge current densities are presented for (d) no, (e) mild, and (f) heavy breakthrough flow regimes. Each setting has been performed triple, and sample-to-sample variation causes some cell voltage differences.

Controlling the gas back-pressure with a needle valve at the outlet resulted in three distinct gas breakthrough regimes. For 20 mbarg or less, there was no breakthrough of gas into the anolyte. Between 20 and 30 mbarg, a mild breakthrough was observed, while for back-pressures >30 mbarg, a heavy breakthrough of gas into the anolyte was observed. These three regimes are depicted in Figure 2a–c.

The influence of these gas breakthrough regimes on cell voltage is presented in Figure 2d–f. When no pressure difference was applied (Figure 2d), the cell supported current densities ≤10 A/m^2^ (12 W/m^2^) with <5% loss in original OCV. But current densities higher than 10 A/m^2^ suffered from higher voltage loss. This can be attributed to the penetrating of water from the catalyst layer. Even though this layer is micrometer thin, the diffusion of H_2_ is too slow to sustain higher current densities.

Applying a pressure difference between 20 and 30 mbarg allowed a mild breakthrough of H_2_ into the anolyte (Figure 2e). The H_2_ pushes the liquid front further toward the catholyte compartment, and the H_2_ reacts with the anolyte at the catalyst surface. Consequently, the cell sustained currents up to 100 A/m^2^ (110 W/m^2^) while losing ≤10% of the anode voltage compared to the OCV. Increasing the back-pressure above 30 mbarg led to heavier H_2_ breakthrough into the anolyte (Figure 2f), resulting in a higher voltage drop at 50 A/m^2^ and a complete loss of voltage at 100 A/m^2^. This heavy breakthrough of H_2_ into the anolyte may cut the KOH supply to the catalyst surface. Thus, it was concluded that a mild breakthrough of H_2_ into the anolyte is imperative in mitigating mass transport limitations and enhancing cell performance.

**Figure 3:**
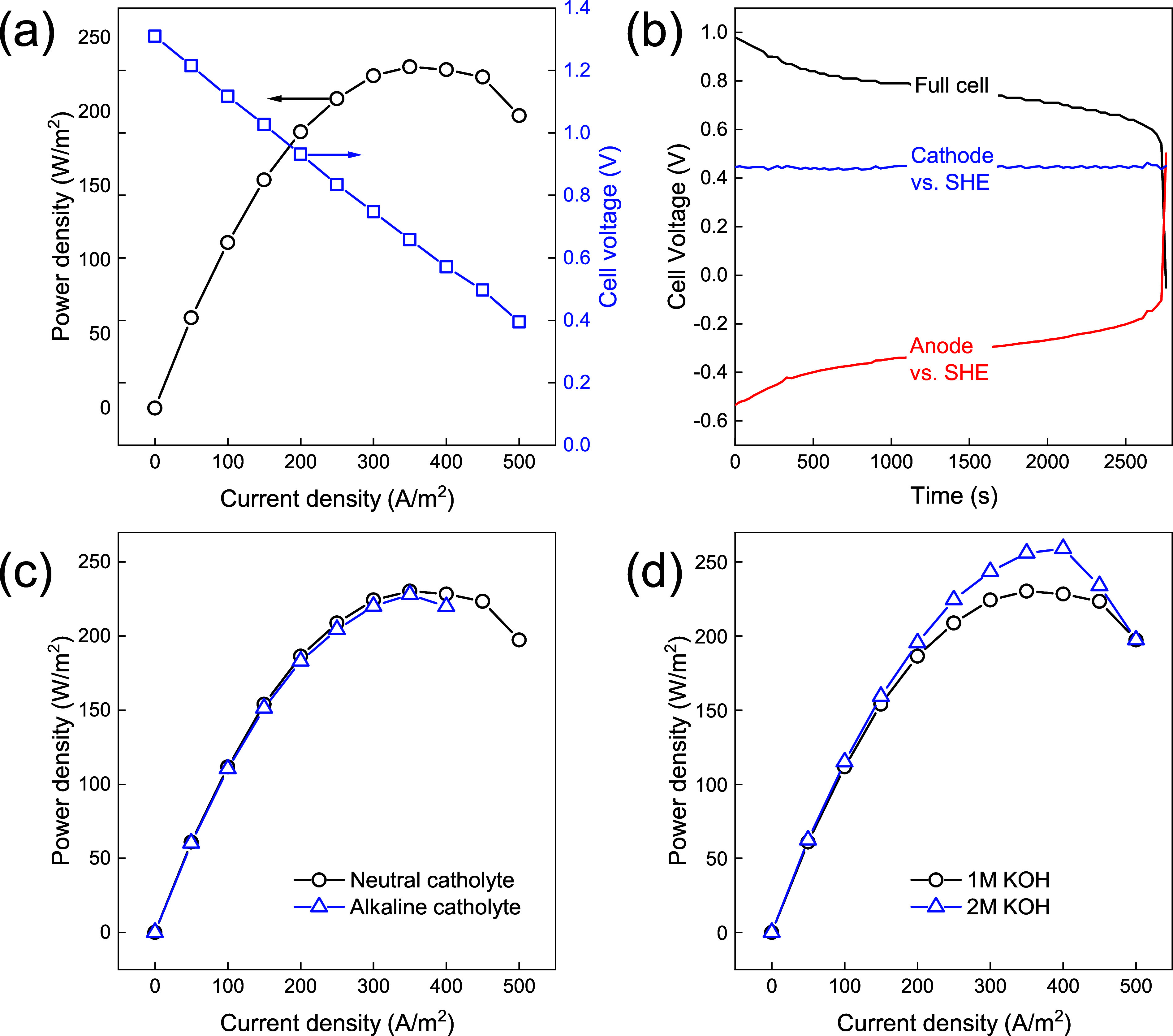
(a) Cell voltage (right—blue) and the corresponding power (left—black) of a full cell as a function of applied current, obtained via LSV. (b) Cell voltage during discharge at 100 A/m^2^ with the contributing cathodic (blue) and anodic (red) half-cell potentials as a function of time. The half-cell potentials are vs SHE. (c) Power curve for cell with a neutral and alkaline catholyte, paired with a 1 M KOH anolyte. (d) Power curve for cell with a neutral 2 M KI + 1 M I_2_ catholyte, paired with 1 and 2 M KOH as an anolyte.

The cell was further characterized in a mild breakthrough regime by performing linear sweep voltammetry (LSV). As shown in Figure 3a, the cell delivers a peak power of 230 W/m^2^ at 350 A/m^2^, significantly higher than the current density sustained by the cell under no and heavy breakthrough regimes. A linear decay in cell voltage points toward dominant Ohmic losses. At 350 A/m^2^, the loss in membranes and electrolytes is estimated to be 290–310 mV (calculation in Supporting Information), which is half of the total voltage loss. This estimate excludes electrical losses in the electrodes and electrical contacts. Thus, the peak power of this nonoptimized system can be nearly doubled by cell design interventions including thinner flow fields, higher concentrations, and more conductive membranes to mitigate Ohmic losses.

While the anolyte in the proposed H_2_–I_2_ cell should remain alkaline to maximize the anodic half-cell potential, the catholyte presents a choice between neutral and alkaline. Because the CEM allows (ideally) only cations to pass and [K^+^] is significantly higher than that of [OH^–^] even under neutral pH, the use of an alkaline anolyte and a neutral catholyte is possible without acid or base crossing over. Hence, even though a pH gradient of 7 units is imposed, the absence of an acid or a base that can cross the CEM renders this cell configuration stable. In the range of current densities evaluated here, cells with a neutral catholyte either perform similarly or better than cells with an alkaline catholyte (Figure 3c), proving that an alkaline catholyte is nonessential for cell performance. In addition, I_2_ oxidizes in alkaline conditions into iodate or periodate, affecting the cathodic half-cell reaction and potentially lowering the energy density. Thus, adding base to the catholyte adds no value.

Regarding the anolyte, a higher concentration of KOH positively correlates with the output power of the cell, as presented in Figure 3d. Doubling anolyte concentration from 1 to 2 M increases the peak power and current density by 10–15%. It highlights the importance of reducing Ohmic resistance in improving the power density of the cell by interventions other than increasing concentrations, such as a minimal or zero gap cell configuration. Also, further engineering of the catalyst loading, membrane thickness, and membrane conductivity will be required to unlock high power densities of the H_2_–I_2_ flow battery.

**Figure 4:**
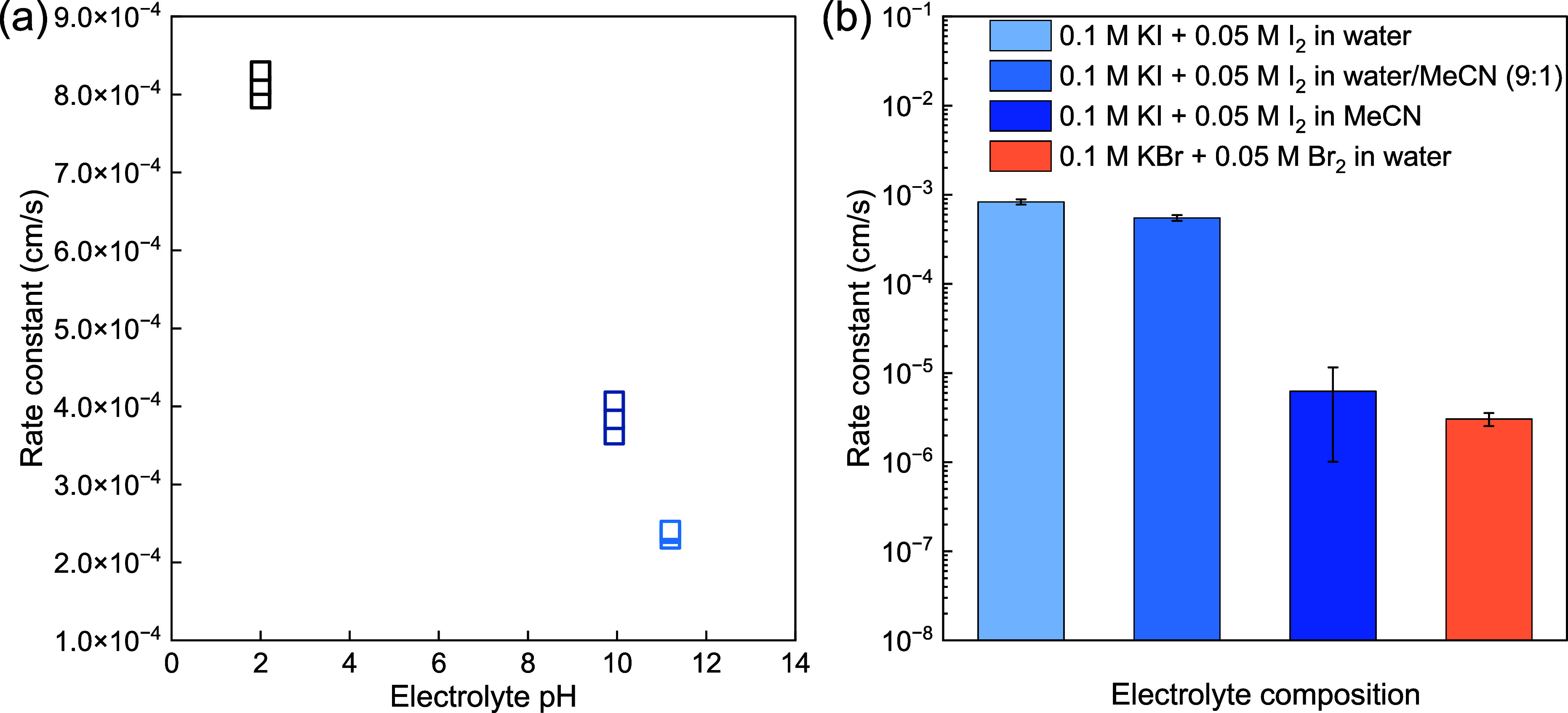
(a) Rate constants obtained from electrical impedance spectroscopy for the iodine half-cell reaction as a function of pH for the same I^–^/I_2_ concentration. (b) Comparison of rate constants for the iodine half-cell reaction with different solvents for the electrolyte and the bromine half-cell reaction in water.

When zooming into the iodine half-reaction at the cathode (eq 1), as shown in Figure 4a, we observe that the kinetics of the reaction, obtained from electrical impedance spectroscopy (see Figure S3), are slightly higher at low pH compared to high pH. This further supports the use of a neutral pH for the catholyte rather than an alkaline condition. The kinetics of the iodine half-reaction are not influenced by the addition of up to 10% MeCN to the KOH solution, as shown in Figure 4b, as the rate constants remain virtually unchanged. A bigger influence is observed when the catholyte is prepared in pure MeCN, which can be expected since it changes the nature of the electrolyte itself to a less polar solvent. The pH of the catholyte with 10% MeCN was equivalent to the electrolyte without MeCN and was measured between 6.5 and 7. Even though the reaction rate constant is slightly decreased at neutral to high pH, the *k*^0^ for I_2_/I^–^ is still 2 orders of magnitude higher than that for Br_2_/Br^–^, further demonstrating the competitiveness of this redox couple to Br_2_/Br^–^, as shown in Figure 4b.

A detailed look at the anode reveals that the losses are larger at the anode than at the cathode; at the start of the discharge at 100 A/m^2^, the anode loses 0.3 V compared to the H_2_/OH^–^ standard potential, while the cathode is within 0.1 V of the I^–^/I_3_^–^ standard potential (Figure 3b). The anode overpotential is substantially higher than in H_2_–Br_2_ flow batteries^35^ and alkaline fuel cells,^36^ which indicates a poor reaction environment. While the cathodic half-cell potential remains constant during discharge, the anodic half-cell potential decays with time until it collapses completely. Such behavior has been observed in literature for diffusion-limited HOR.^32^ Because H_2_ is fed continuously from a gas bottle, it should be either the OH^–^ from the anolyte that is limited over time or a change in electrode wetting that causes H_2_ diffusion limitation over time. To study these effects, we performed charge–discharge cycles.

**Figure 5:**
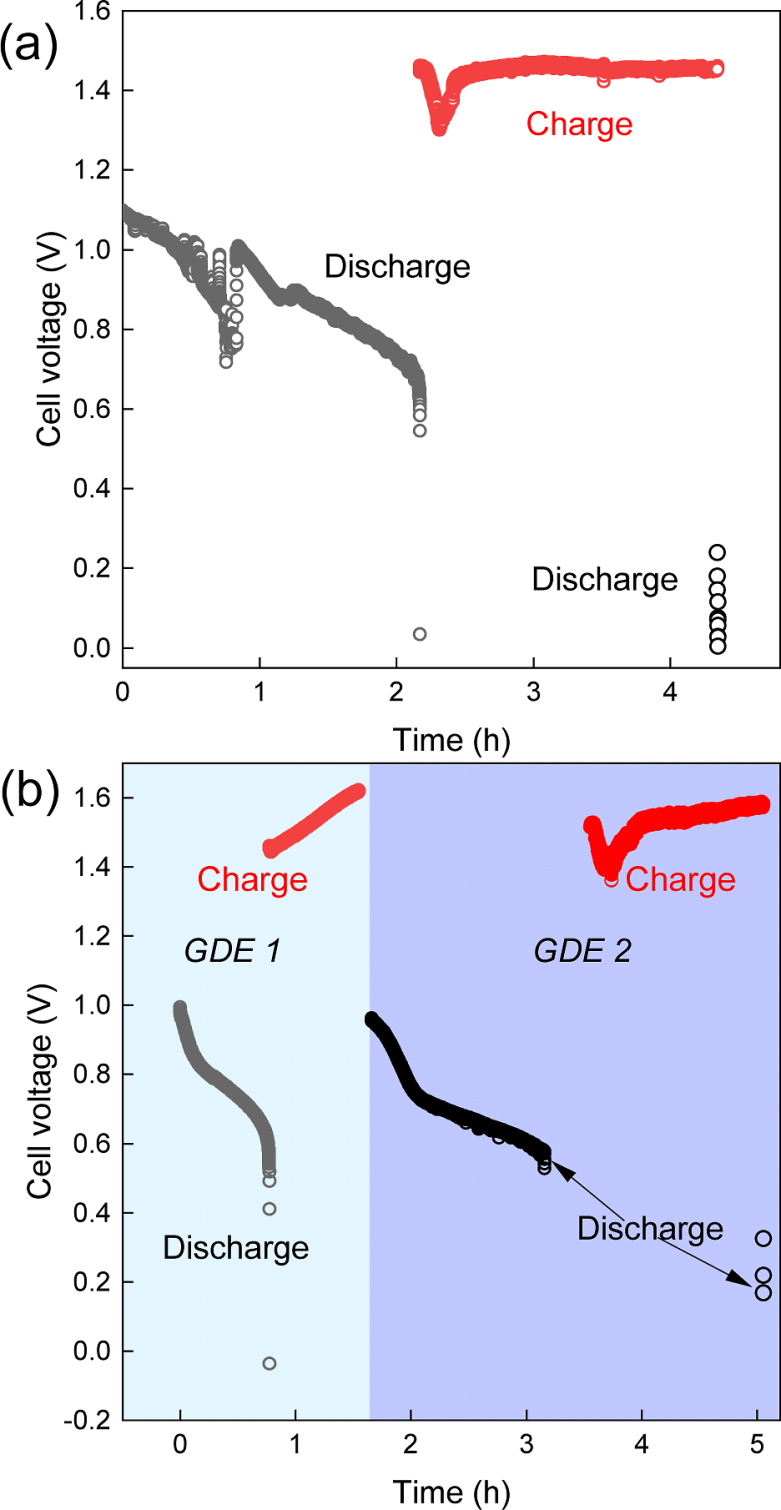
(a) Cell voltage as a function of time, obtained for a H_2_–I_2_ cell at 100 A/m^2^, without changing the GDE at the anode. (b) Cell voltage as a function of time, obtained for a H_2_–I_2_ cell at 100 A/m^2^, for two different GDEs, depicted with distinct color shades in the graph.

The cell was discharged for 2.1 h and subsequently charged at 100 A/m^2^ for the same duration, delivering a voltage efficiency of 61% (Figure 5a). The cell voltage during the second discharge collapses to 0 V within seconds and correlates with a positive anodic half-cell potential. This is attributed to GDE degradation under charging, which is proven from the successful second discharge cycle performed after GDE was replaced after the first full cycle (Figure 5b). With fresh GDE, without replacing the electrolytes, the cell can discharge again at 100 A/m^2^. When attempting a second discharge with GDE 2, after the battery was charged with this second GDE, the cell voltage collapsed to 0 V within seconds.

Another experiment in which the cell was run only in discharge mode with changing electrolytes at the end of every discharge is presented in Figure S5. While the subsequent discharging reduces the energy output (duration of second discharge < first discharge), it is unlike the complete loss of electrochemical activity observed after a charge step, as presented in Figure 5. It proves further that the charging step accelerates the loss of electrochemical activity in the GDE. The Freudenberg H23C6 GDE is known for its degradation upon applying strong reduction potentials.^34,37^ A material screening study would be required to identify gas diffusion electrodes with better stability against alkaline and reducing conditions. We believe that materials from the field of AEM water electrolysis and alkaline fuel cells can provide useful leads for such a material.

**Figure 6:**
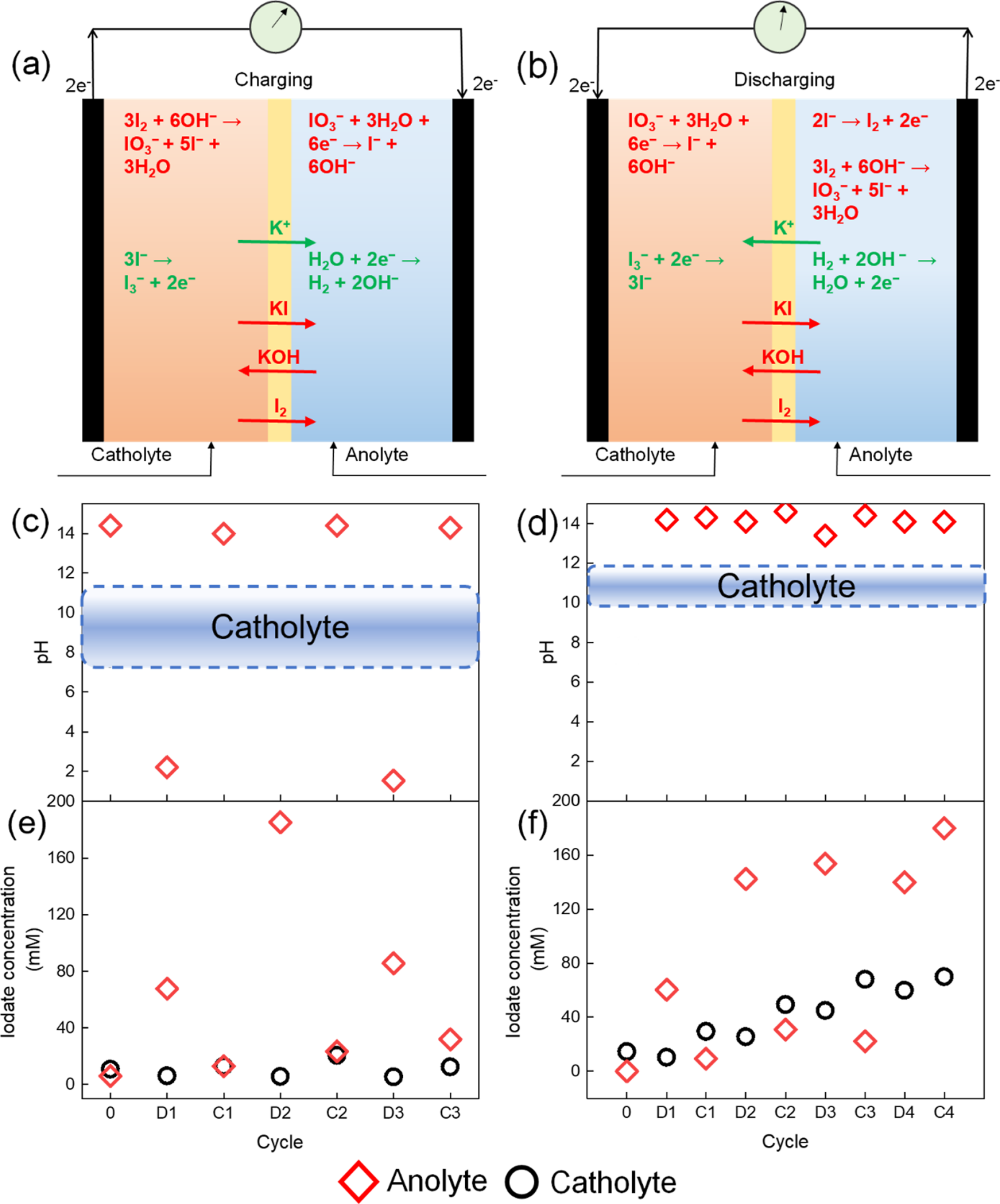
Electrolyte composition during cycling. To focus on the crossover, no H_2_ was fed to the cell, and as a result, oxygen evolution occurred at the anode during discharge. The cell was cycled at 500 A/m^2^, with 2 h charge and discharge cycles, and the composition of the electrolytes was measured after every (dis)charge cycle. Desired (green) and undesired (red) ion transport between the catholyte and anolyte during (a) charging and (b) discharging of a H_2_–I_2_ RFB. (c) Change in pH and (e) iodate concentration of the anolyte (red) and the catholyte for the H_2_–I_2_ battery with 1 M KOH as the anolyte. (d) Change in pH and (f) iodate concentration of the anolyte (red) and the catholyte (black) for the H_2_–I_2_ battery with 2 M KOH as the anolyte.

Finally, the ion crossover across the CEM was investigated in a two-compartment cell, without the GDE and the gas compartment, to understand the cyclic changes in pH and ionic composition of the electrolytes. The hypothesized ion transport and reactions (green—wanted; red—unwanted) during (dis)charge are presented in Figure 6a,b. While the movement of K^+^ between compartments and the movement of I^–^/I_3_^–^ and OH^–^/H_2_ toward the cathode/anode is desirable, the movement of iodine species and OH^–^ between compartments is undesirable since it leads to pH change and iodate (IO_3_^–^) formation, as shown in Figure 6c,d.

Ideally, there should be no transport of OH^–^, I^–^, I_3_^–^, H^+^, and I_2_. These three anions should be rejected by Donnan exclusion in the CEM, while the concentrations of H^+^ and I_2_ should be small due to the pH 7–14 conditions and the complexation of I_2_ with I^–^ into I_3_^–^. Avoiding crossover of all of these species would automatically prevent the formation of iodate. If crossover occurs due to nonideal selectivity, IO_3_^–^ can be formed via a combination of I_2_ with OH^–^ (see Figure 6a,b). Therefore, the formation of IO_3_^–^ is an insight into OH^–^ diffusion as well as available I_2_.

The anolyte pH shows an extreme fluctuation (Figure 6c) because the [OH^–^] in the anolyte was matched too closely to the discharge time, which induces overdischarging of the anolyte. During charging, KOH is regenerated at the anode, resulting in an anolytic pH rise, confirmed in Figure 6c. The catholyte pH also changes with the direction of current by ±1 unit but remains within a band of values, as shown in Figure 6c. This corresponds to a [OH^–^] fluctuation in the catholyte of maximum 1 mM, remaining steady for 12 h of cycling.

The KOH depletion at the anode is eliminated by changing the anolyte to a concentration of 2 M KOH, without changing the catholyte, as shown in Figure 6d. This results in stable anodic pH. However, doubling KOH concentration increases the gradient between the anolyte and catholyte, and as a result, the average catholyte pH increases in comparison to the anolyte configuration with 1 M KOH, as shown in the shaded region of Figure 6d. Nevertheless, the absolute [OH^–^] in the catholyte is still <10 mM, which corresponds to <0.3% of the charge transfer over 8 h of charging. This is comparable (or slightly smaller) to the expected crossover based on the initial concentrations and membrane selectivity (see Supporting Information).

Another crossover of interest is that of iodide and iodine species and the associated side reaction of I_2_ to IO_3_^–^. As hypothesized, [IO_3_^–^] in the catholyte increases during charge and decreases during discharge (Figure 6e,f), following the pattern of [I_2_], which is highest after charging. For a 1 M KOH anolyte, the [IO_3_^–^] in the catholyte remains steady (<20 mM) over multiple cycles. Thus, loss of active species due to side reaction is contained and reversed in the catholyte. This is not the case for configuration with 2 M KOH as the anolyte since the [IO_3_^–^] rises steadily in the catholyte between cycles. This accumulation is attributed to the increased OH^–^ crossover into the catholyte because of the increased [OH^–^] gradient. However, the cyclic rise and fall in [IO_3_^–^] implies that even at increased crossover, IO_3_^–^ can be reversed, forming I^–^, according to the reaction given in panels (a) and (b).^38,39^ A higher complexation of I_2_ will prevent the capacity loss from side reactions.

Iodate is also (reversibly) formed in the anolyte, as can be seen in Figure 6e,f. The I^–^ crosses over to the anolyte because of the concentration gradient. During discharge, I^–^ oxidizes to I_2_, which reacts with OH^–^ to form IO_3_^–^. During charging, the [IO_3_^–^] decreases, implying that the rate of the IO_3_^–^ decomposition reaction, together with I^–^ and IO_3_^–^ migrating back to the anolyte, exceeds the concentration gradient-driven diffusion. The [IO_3_^–^] in the anolyte remains within the same range for 1 and 2 M KOH anolytes, implying that the formation of IO_3_^–^ is limited by [I_2_] and not by the anolyte [OH^–^]. An exception is [IO_3_^–^] during the fourth charge cycle, which may be related to the higher [IO_3_^–^] in the catholyte, reducing the diffusion to the catholyte. To mitigate the loss of I^–^ from the catholyte, I_2_ may be recovered from the anolyte once IO_3_^–^ converts to I^–^ during charging or a more selective cation exchange membrane would reduce the necessity for electrolyte regeneration.

## Conclusions

In summary, we investigate a H_2_–I_2_ redox flow battery, combining the potential of fast iodide/iodine kinetics with its high solubility and the high OCV (1.37 V) in an alkaline anolyte. A GDE is proposed to reduce the mass transport limitation at the anode and delivers a maximum power of 230 W/m^2^. A mild H_2_ breakthrough into the anolyte via the GDE is found critical for sustaining high current densities. Furthermore, the power output of the cell is independent of the pH of the catholyte, which allows stable operation with an alkaline H_2_ electrode and a neutral iodine-based electrode. We also observe that the GDE loses its electrochemical activity during the charging step, resulting in voltage loss during the discharge that follows. To further increase the power densities to industrially relevant figures, a more stable GDE is required, as well as engineering the membrane conductivity, compartment thickness, and catalyst deposition. And finally, while the crossover of OH^–^ into the catholyte can be minimized when using 1 M KOH as the anolyte, crossover of I^–^ into the anolyte leads to reversible iodate formation and requires further optimization to make the H_2_–I_2_ redox flow battery a practical energy storage system.

## Supporting Information Available

Experimental details, results for a two-compartment setup, estimation of H^+^ and OH^–^ crossover vs K^+^ transport, and estimation of resistive losses in a H_2_–I_2_ cell (PDF)
