# Supplementary material for: Balancing pH and Pressure Allows Boosting Voltage and Power Density for a H2–I2 Redox Flow Battery
Source: ACS Appl Energy Mater. 2024 Dec 24;8(1):631–9. doi: 10.1021/acsaem.4c03032 (PMC11733921; doi:10.1021/acsaem.4c03032)
Supplement: Supplementary file 1 — ae4c03032_si_001.pdf [file ae4c03032_si_001.pdf]

Supporting Information

for

Balancing pH and pressure allows boosting voltage and power density for a H_2-I_2 redox flow battery

Kaustub Singh,[1*] Ameya Bondre,[1*] Kostadin V. Petrov,[1] David A. Vermaas[1#]

Department of Chemical Engineering, Delft University of Technology, Van der Maasweg 9, 2629 HZ Delft, The Netherlands. [#] Correspondence to D.A.Vermaas@tudelft.nl

Experimental details

A two-compartment Microcell (ElectroCell, Denmark) module was used as the base for this cell, using PTFE flow channels. The total active area in the cell was 10 cm^2. The Pt catalyst was deposited over the electrode using ultra-high vacuum sputter deposition (AJA International Inc.) of Pt nanoparticles (MaTeck, 99.9 % purity) with a 50 W power supply in a 20 sccm inert Ar atmosphere at a rate of 2.44 Å/s. The thickness of the catalyst layer was 100 nm.

To provide a reference for the GDE-based setup, we also tested a two-compartment control experiment. The H_2 in this experiment was sparged into the anolyte reservoir and carried into the cell in dissolved state. Schematic illustrating this system is presented in **Figure S1(a)**.

Experiments performed to characterize the performance of the three-compartment H_2 – I_2 full cell can be divided into the following sections

The correlation between the gas backpressure and the gross power output of the cell was studied by varying the position of the needle valve (open to close) at the gas outlet, and the applied current. Three distinct H_2 flow regimes, namely - no breakthrough, mild breakthrough, and heavy breakthrough were identified between pressure difference of 18 – 35 mBarg between the gas and the liquid outlets. For all three regimes, the cell was

discharged with an PGTSTAT302N potentiostat (Metrohm, Autolab) in galvanostatic mode at 10, 50, and 100 A/m^2 for a duration of 10 minutes.

Following the pressure-tuning of the cell, a linear sweep voltammetry (LSV) was performed in the current range of 0 to 500 A/m^2, with a current-step of 50 A/m^2 and a time-step of 1 minute. The LSV was also performed for two different anolyte concentrations (1M KOH and 2M KOH) to correlate cell power output with anolyte concentration. Finally, an LSV was performed on the three-compartment cell with an alkaline catholyte, prepared in 1 M KOH (same as the standard anolyte concentration) to help understand the influence of catholyte pH, if any, on cell output power.

In addition to the LSV, the cell was cycled in three-compartment configurations at 100 A/m^2 to investigate the charge-discharge behaviour in a wider SOC window. Also, the crossover of species between the catholyte and the anolyte was performed in a two-compartment setup, cycling at a 500 A/m^2.

Two-compartment setup

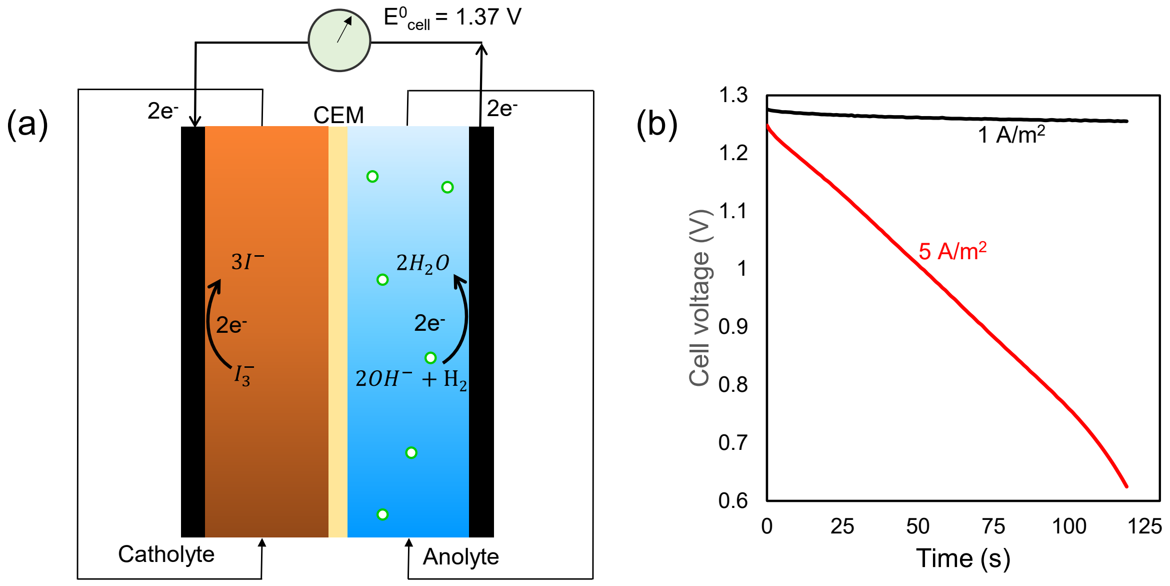

Figure S1: (a) Two-compartment setup consisting of KI + I_2 catholyte and KOH + H_2 anolyte, separated by a CEM. The H_2 is fed to the system by sparging it directly into the anolyte. (b) Voltage of the two-compartment cell as a function of time, obtained for a current of 1 and 5 A/m^2.

Estimation of H^+ and OH^- crossover vs K^+ transport

To estimate the crossover of H^+ and OH^-, in relation to the charge transport via K^+, we can use the membrane selectivity and concentrations of the anolyte and catholyte. At default conditions, the concentrations of anolyte and catholyte are given in **Table S1**.

Table S1: Initial concentrations for the $H_2 - I_2$ flow cell

	$[K^+]$	$[H^+]$	$[OH^-]$
Anolyte	1.0 M	10^{-14} M	1.0 M
Catholyte	2.0 M	10^{-7} M	10^{-7} M

The selectivity for H^+ transport vs K^+ transport in typical cation exchange membranes 1.5-2 (we assume an average value of 1.75 for this calculation).[1] The counter-ion vs co-ion selectivity for cation exchange membranes is typically in the order of magnitude of 100.[2] That means we can expect the following transport numbers t as provided in **Table S2**.

Table S2: Transport numbers based on initial concentrations (Table S1) and estimated membrane selectivities.

	t_{K+}	t_{H+}	t_{OH-}
Charging	0.995	$9\cdot10^{-8}$	0.005
Discharging	0.999999998	$2\cdot10^{-14}$	$2\cdot10^{-9}$

Hence, the expected direct crossover of H^+ is negligible. A slight crossover of OH^- can be expected, of 0.5% of the charge transfer, during the charging phase.

Estimation of resistive losses in an $H_2 - I_2$ cell

Resistance of the cell is calculated using the following equation [3]:

$$R_{\text{cell}} = \left(\frac{R_{\text{AEM}}}{1-\beta} + \frac{R_{\text{CEM}}}{1-\beta} + \frac{d_a}{\epsilon^2\phi_a} + \frac{d_c}{\epsilon^2\phi_b}\right) \quad (S1)$$

Where R_{AEM}, R_{CEM} are the resistances of the AEM and CEM, respectively, d is the intermembrane distance (or the thickness of the flow frames used for fluid flow), β is the flow frame shadow factor, ϵ is the flow frame porosity, and Φ is the conductivity for catholyte

(c) and anolyte (a). Values for these parameters is given in **Table S3** below. The membrane resistance values were sourced from supplier information website (Fumatech, Germany).

Table S3: Parameters used to calculate resistance of a three compartment $H_2 - I_2$ flow cell

Parameter	Value
R_{AEM} (mOhm.cm^2)	4
R_{CEM} (mOhm.cm^2)	3.5
d (mm)	2
Φ_c – *neutral* (S/m)	16.9
Φc – *alkaline* (S/m)	23.3
Φ_a (S/m)	6.35
β	0.5
ϵ	0.7

The cell resistance from equation (S1) can be multiplied by the applied current density to convert the resistive losses in V. For 350 A/m^2 , the current density at which peak power was measured, resistive voltage loss amounts to 0.309 V for the cell with neutral catholyte and 0.286 V for the cell with alkaline anolyte. These losses are 46 and 50 % of the total cell voltage (0.62 V) obtained at 350 A/m^2.

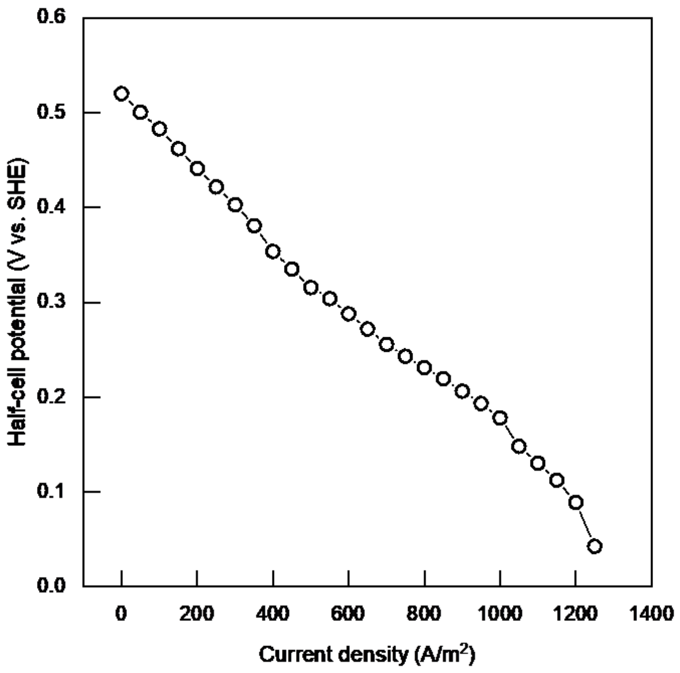

Figure S2: Half-cell potential vs. SHE as a function of current obtained via LSV on the cathodic half of the H_2-I_2 cell.

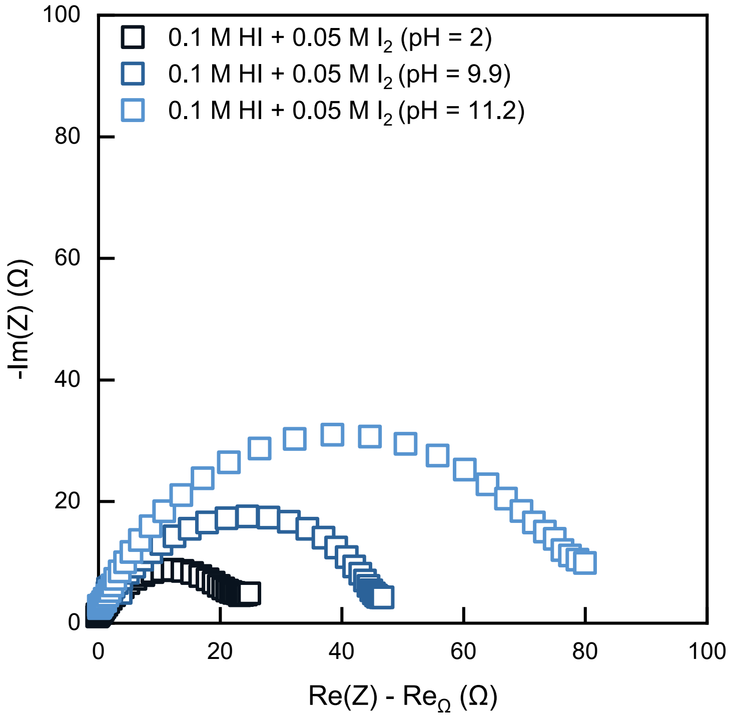

Figure S3: Impedance spectra of the iodine half-cell reaction as a function of pH for the same I^-/I_2 concentration. Reaction rate transfer coefficients were calculated from the charge transfer resistance obtained from these Nyquist plots.

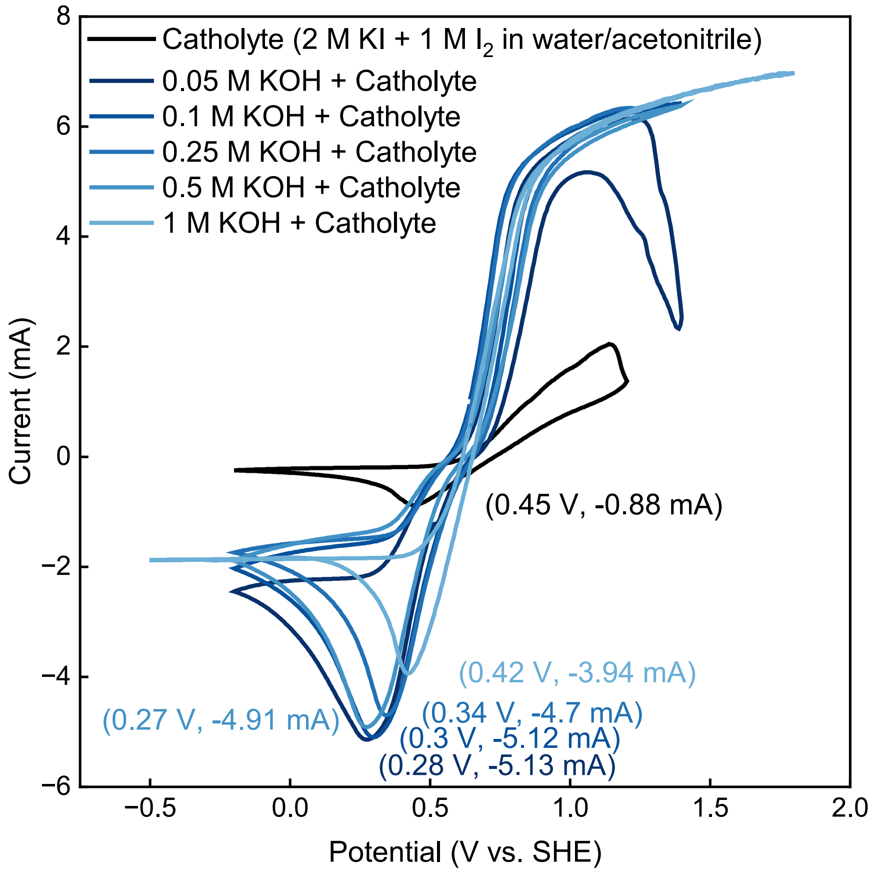

Figure S4: Cyclic voltammograms of the catholyte (2 M KI + 1 M I_2 in water/acetonitrile mixture) as a function of KOH concentration.

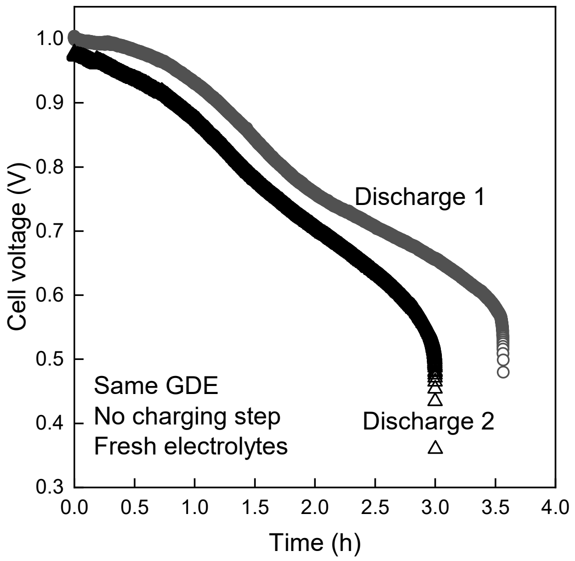

Figure S5: Cell voltage as a function of time, obtained for a $H_2 - I_2$ cell at 100 A/m^2 with fresh electrolytes, without changing the GDE at the anode. The electrolytes were not charged in between the two discharge steps and instead, fresh electrolytes were used during the two discharge operations.

REFERENCES

[1] Miyoshi, H., Yamagami, M., Chubachi, M., & Kataoka, T. (1994). Characteristic coefficients of cation-exchange membranes for bivalent cations in equilibrium between the membrane and solution. *Journal of Chemical and Engineering Data*, *39*(3), 595-598.

[2] Dischinger, S. M., Miller, D. J., Vermaas, D. A., & Kingsbury, R. S. (2024). Unifying the Conversation: Membrane Separation Performance in Energy, Water, and Industrial Applications. *ACS ES&T Engineering*, *4*(2), 277-289.

[3] Ranade, A., Singh, K., Tamburini, A., Micale, G. & Vermaas, D. A. Feasibility of Producing Electricity, Hydrogen, and Chlorine via Reverse Electrodialysis. *Environ. Sci. Technol.* **56**, 16062–16072 (2022).
